# Enteroids to Study Pediatric Intestinal Drug Transport

**DOI:** 10.1021/acs.molpharmaceut.4c00339

**Published:** 2024-09-16

**Authors:** Eva J. Streekstra, Marit Keuper-Navis, Jeroen J. M. W. van den Heuvel, Petra van den Broek, Martijn W. J. Stommel, Willem de Boode, Sanne Botden, Sander Bervoets, Luke O’Gorman, Rick Greupink, Frans G. M. Russel, Evita van de Steeg, Saskia N. de Wildt

**Affiliations:** † Division of Pharmacology and Toxicology, Department of Pharmacy, Radboud University Medical Center, Nijmegen 6525GA, The Netherlands; ‡ Department of Metabolic Health Research, Netherlands Organization for Applied Scientific Research (TNO), Leiden 2333BE, The Netherlands; § Division of Pharmacology, Utrecht Institute for Pharmaceutical Sciences (UIPS), Utrecht University, Utrecht 3584 CS, The Netherlands; ∥ Department of Surgery, Radboud University Medical Center, Nijmegen 6525GA, The Netherlands; ⊥ Department of Pediatrics, Division of Neonatology, Radboud University Medical Center, Amalia Children’s Hospital, Nijmegen 6525GA, The Netherlands; # Department of Surgery, Amalia Children’s Hospital, Radboud University Medical Center, Nijmegen 6525GA, The Netherlands; ¶ Radboudumc Technology Center for Bioinformatics, Department of Medical BioSciences, Radboud University Medical Center, Nijmegen 6525GA, The Netherlands; ∇ Department of Intensive Care, Radboud University Medical Center, Nijmegen 6525GA, The Netherlands; ○ Department of Neonatal and Pediatric Intensive Care, Erasmus MC Sophia Children’s Hospital, Rotterdam 3015 GD, The Netherlands

**Keywords:** intestine, intestinal organoids, enteroids, pediatrics, Ussing chamber, pharmacokinetics, drug transporters

## Abstract

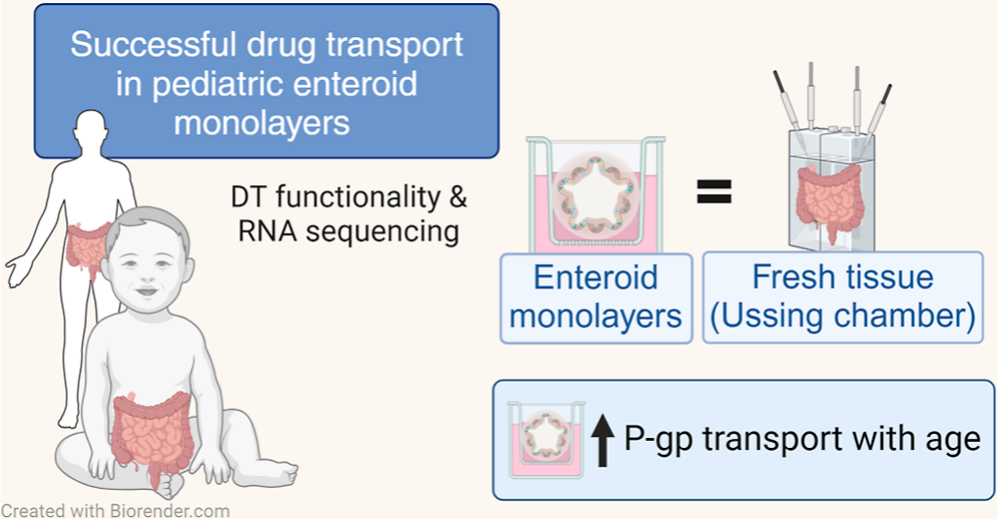

Intestinal maturational changes after birth affect the pharmacokinetics (PK) of drugs, having major implications for drug safety and efficacy. However, little is known about ontogeny-related PK patterns in the intestine. To explore the accuracy of human enteroid monolayers for studying drug transport in the pediatric intestine, we compared the drug transporter functionality and expression in enteroid monolayers and tissue from pediatrics and adults. Enteroid monolayers were cultured of 14 pediatric [median (range) age: 44 weeks (2 days–13 years)] and 5 adult donors, in which bidirectional drug transport experiments were performed. In parallel, we performed similar experiments with tissue explants in Ussing chamber using 11 pediatric [median (range) age: 54 weeks (15 weeks–10 years)] and 6 adult tissues. Enalaprilat, propranolol, talinolol, and rosuvastatin were used to test paracellular, transcellular, and transporter-mediated efflux by P-gp and breast cancer resistance protein (BCRP), respectively. In addition, we compared the expression patterns of ADME-related genes in pediatric and adult enteroid monolayers with tissues using RNA sequencing. Efflux transport by P-gp and BCRP was comparable between the enteroids and tissue. Efflux ratios (ERs) of talinolol and rosuvastatin by P-gp and BCRP, respectively, were higher in enteroid monolayers compared to Ussing chamber, likely caused by experimental differences in model setup and cellular layers present. Explorative statistics on the correlation with age showed trends of increasing ER with age for P-gp in enteroid monolayers; however, it was not significant. In the Ussing chamber setup, lower enalaprilat and propranolol transport was observed with age. Importantly, the RNA sequencing pathway analysis revealed that age-related variation in drug metabolism between neonates and adults was present in both enteroids and intestinal tissue. Age-related differences between 0 and 6 months old and adults were observed in tissue as well as in enteroid monolayers, although to a lesser extent. This study provides the first data for the further development of pediatric enteroids as an in vitro model to study age-related variation in drug transport. Overall, drug transport in enteroids was in line with data obtained from ex vivo tissue (using chamber) experiments. Additionally, pathway analysis showed similar PK-related differences between neonates and adults in both tissue and enteroid monolayers. Given the challenge to elucidate the effect of developmental changes in the pediatric age range in human tissue, intestinal enteroids derived from pediatric patients could provide a versatile experimental platform to study pediatric phenotypes.

## Introduction

Pharmacokinetics (PK) in the pediatric population is profoundly impacted by the dynamic process of maturation in early life. During growth, children undergo several physiological changes that impact drug absorption, distribution, metabolism, and excretion (ADME). This can have major implications for drug dosing, as these changes can affect drug safety and efficacy.^1^ While liver and kidney maturation clearly impacts drug transporter (DT) abundance and/or functionality,^2−5^ less is known about ontogeny-related DT expression and functionality in the intestine.

The intestine is a dynamic organ serving as a selective barrier that regulates uptake of nutrients, while simultaneously limiting entry of pathogens and exogenous compounds such as drugs. Enterocytes are the absorptive cells in the epithelial barrier that express uptake and efflux membrane transporters, facilitating the absorption of compounds into the circulation or excretion of compounds back into the intestinal lumen.^6^ Efflux of a drug back into the intestinal lumen limits its bioavailability; therefore, systemic exposure is severely impacted by this process. P-Glycoprotein (P-gp, *ABCB1*) and breast cancer resistance protein (BCRP, *ABCG2*) are the major efflux transporters involved in intestinal drug transport. Proteomics data shows lower abundance of most intestinal transporters in the intestine of 0–2 year olds compared to adults, but evidence is still scarce.^7^

Studying PK profiles in the pediatric population is challenging due to ethical and logistical issues, which limit tissue sampling and invasive procedures. Hence, there is a need for human in vitro models to study age-related changes in drug ADME and enhance our understanding of pediatric PK. Currently applied in vitro models for intestinal absorption are either limited in PK functionalities (e.g., Caco-2, HT-29) or limited in availability, e.g., when dependent on leftover human material from surgeries.^8^ With intestinal tissue, protein functionality (using Ussing chamber), proteomics, or RNA expression studies can be performed. However, the use of tissue comes with several challenges, including tissue availability (number and size of tissue samples or tissue quality) as well as methodology. For example, conducting functional studies with the Ussing chamber is labor intensive and challenging in terms of tissue handling and preparation. Tissue-derived human intestinal organoids (hereafter termed enteroids) could help to overcome these limitations. Enteroids can be isolated from residual intestinal surgical material, are therefore patient-specific, and can be maintained in culture for up to months.^9^ Initially, enteroids grow in a self-organizing 3D formation with a hollow inside representing the luminal side of the intestine; as a result, the lumen is inaccessible for drug transport studies. To mimic drug absorption via the oral route, enteroids can be cultured in 2D monolayer formation on a permeable membrane.^10^ In this way, enteroids provide an opportunity to study drug transport in the intestine.

Recent research that utilizes enteroids derived from pediatric intestinal tissue has indicated that methylation patterns specific to the original donor’s age persist during in vitro culture in 4–15 year olds, while fetal-derived enteroids (8–12 weeks gestational age) changed and matured toward more adult methylation patterns during in vitro culture.^11,12^ Methylation patterns play an essential role in governing physiological pathways in cells. Hence, enteroid in vitro models may hold the potential to capture age-specific biological features, such as ADME gene expression in pediatric enteroids but perhaps also for neonates. Previously, enteroids have been used to study disease processes and drug efficacy in pediatrics including necrotizing enterocolitis and cystic fibrosis.^13,14^ Human enteroid monolayers derived from infants have shown to display characteristics of an immature gastrointestinal epithelium (in line with in vivo observations) compared to adults.^15^

To the best of our knowledge, a systematic comparison between pediatric enteroids and pediatric tissue considering DT expression and functionality is still lacking. Therefore, we provide the first data set to verify the capabilities of enteroid monolayers to study DT in pediatric donors. We do this by providing a direct comparison of drug transport mechanisms (paracellular, transcellular, P-gp, and BCRP) in enteroid monolayers and intestinal tissue of various age groups. Apparent permeability (*P*_app_) values of the model compounds enalaprilat, propranolol, talinolol, and rosuvastatin determined in pediatric and adult tissues were compared with *P*_app_ values derived in enteroid monolayers. Gene expression analysis was performed to compare the age-related expression of ADME-related genes in enteroids and their representative tissues via RNA sequencing.

## Methodology

Human intestinal surgical left over tissue has been collected and used for fresh tissue Ussing chamber experiments as well as enteroid isolation. Drug transport mechanisms were determined across tissue and enteroid monolayer barriers. Next to that, fresh tissue and enteroid monolayers were used for RNA sequencing. Supporting Information Table 2 indicates which donor tissue was used for Ussing chamber, enteroid monolayers, and RNA analysis. Information regarding the composition of buffers and culture medium can be found in the Supporting Information Methods Table 1.

### Human Tissue

Surgical leftover (mid to terminal) ileum tissues from pediatric patients were obtained during surgeries at Radboud university medical center (Radboudumc), Nijmegen, The Netherlands, from 2020 to 2023. Data from 3 out of 11 Ussing chambers performed were taken from a previous publication by Streekstra and Kiss et al., 2022.^16^ Only these experiments were taken, as donors were included in the same time frame and experiments were all performed by the same individuals in the lab. The latter is to reduce variation caused by execution of the methodology. Intestinal tissue proximal from the stoma ending was used when possible, as this has still been exposed to luminal compounds in contrast to the tissue distal to the stoma. For pediatric inclusions, we obtained informed consent from parents/legal guardians and/or children for the use of left over material and clinical data access. Radboudumc Medical Ethics Board waived the need for formal ethics approval according to the Dutch Law on Human Research for pediatric tissues.

Adult ileum tissue data were derived from previous publications on drug transport in jejunum and ileum enteroids (manuscript submitted). Tissue was obtained from (hemi)colectomy surgeries. For the adults, no informed consent was needed for the use of anonymous left-over material for research purposes, following the Dutch Code of Conduct for Responsible Use. Experiments were all performed in the same time frame between 2020 and 2023 and the same research team.

Tissues were transported to the lab in ice-cold Krebs buffer as soon as possible after surgical resection (within 15 min). Tissue was cleaned and undone from the basolateral muscular layers and connective tissue. Part of the tissue was immediately used for Ussing chamber experiments, another part was stored at 4 °C in supplemented Williams’ E storage buffer for enteroid isolation, and a final part of tissue was snap frozen for RNA analysis (Supporting Information Table 1).

## Enteroid Culture

### 3D Culture

Methodology was based on previously published protocols from STEMCELL IntestiCult.^17−20^ In short, intestinal crypts were isolated within 24 h after surgical resection of the tissue to establish enteroids.^21^ Mucosal layers were undone from serosal layers, cut in 2–3 mm^2^ pieces, and washed thoroughly in wash buffer. Tissue pieces were incubated for 1 h in crypt releasing solution, after which fractions were collected in collection buffer. Obtained crypts were diluted in 70% Matrigel and plated in 30 μL droplets in 48 well plates, covered with 300 μL organoid growth medium (OGM). Every 2–3 days, culture medium was refreshed. Cultures were passaged after 5–10 days.

### 2D Enteroid Monolayers

Enteroid monolayer procedures were based on STEMCELL IntestiCult and previously published protocols.^22−25^ Enteroid monolayers were seeded on clear membrane inserts (6.5 mm, 0.4 μm pore size) and coated with collagen type 1. After 5–10 days of enteroid culture, 3D enteroids were disrupted to single cell with TripLE and seeded 1 × 10^5^ cells/well in OGM. Basolateral medium was added directly after seeding. When the confluent monolayer was confirmed by microscopy, the OGM was replaced with organoid differentiation medium for 5 days. Medium was changed every 2–3 days.

### Bidirectional Drug Transport in Monolayers

After 5 days of enteroid monolayer differentiation, bidirectional drug transport studies were performed in the apical to basolateral (A-to-B) or basolateral to apical (B-to-A) direction. Transepithelial electrical resistance (TEER) measurements were performed before and after the experiments in a transport buffer. The drug cocktail (see section Drug cocktail) prepared in transport buffer was added on either the apical or basolateral side of the monolayer. Fluorescein-dextran 4 kDa (FD4, 50 μM) was added to the apical buffer of each insert to monitor barrier integrity during the experiment. For FD4 transport, a *P*_app_ cut off value of <1 × 10^–6^ was used.^26^ Samples (50 μL) were taken at the acceptor side at 30, 60, and 120 min and the donor side at 0 and 120 min to determine apparent permeability through the monolayers. During the experiment, monolayers were placed on a rocker (70 rpm) in a humidified incubator at 37 °C.^27,28^ Cell lysates were collected for RNA analysis.

### Bidirectional Drug Transport in Tissue (Ussing Chamber)

Ussing chamber experiments were described in detail by Streekstra and Kiss at al., 2022.^16^ In short, intestinal mucosal pieces were put in Ussing chamber sliders (0.2 cm^2^ for pediatrics, 0.71 cm^2^ for adults) and mounted between two Ussing chamber halves. The experiment was performed at 37 °C with continuous carbonated buffer. During the experiment, tissue viability was monitored using electrophysiology measurements. After stabilization, buffer was replaced with transport buffer containing the drug cocktail of interest to start the bidirectional transport assay (see section Drug Cocktail). Every 15 min, samples of 50 μL were taken on the acceptor and donor side of the tissue up to 2 h and replaced with fresh buffer.

### Drug Cocktail

To compare the drug transport in enteroid monolayers with fresh intestinal tissue, we used the exact same drug cocktail in both models. Passive transport was monitored with the paracellular and transcellular transport markers enalaprilat (10 μM) and propranolol (10 μM), respectively.^29−31^ The substrates talinolol (2 μM) and rosuvastatin (5 μM) were used for the efflux transporters P-gp and BCRP, respectively.^32−35^ To determine the apparent permeability (*P*_app_) of the substrates across the tissue and enteroid barrier, drug concentrations in the acceptor and donor compartments were determined by liquid chromatography tandem mass spectrometry (LC–MS/MS).

### Liquid Chromatography Tandem Mass Spectrometry

Compound concentrations in each sample were determined by LC–MS/MS by the previously described methodology.^16^ In short, d5-enalaprilat and labetalol were used as internal standards. An Acquity UPLC (Waters, Milford, MA, USA) was coupled to a Xevo TQ-S (Waters) triple quadrupole mass spectrometer. A HSS T3 analytical column (1.8 μm; 100 × 2.1 mm, Acquity UPLC, Waters, Ireland) was used to separate the compounds.

### Gene Expression Analysis

RNA-seq analysis was performed on snap frozen intestinal tissue and differentiated enteroid monolayers cultured on permeable inserts. The RNeasy Mini kit (Qiagen) was used according to the manufacturer’s protocol to isolate mRNA. RNA sequencing libraries were prepared using the KAPA RNA HyperPrep kit with RiboErase (human/mouse/rat [HMR]) (Kapa Biosystems). This involved various steps: oligonucleotide hybridization and rRNA depletion, rRNA depletion cleanup, DNase digestion, DNase digestion cleanup, and RNA elution. Fragmentation and priming occurred at 94 °C for 6 min. First-strand synthesis, second-strand synthesis, and A-tailing followed standard protocols. Adapter ligation utilized a 1.5 μM stock (NEXTflex DNA barcodes; Sanbyo), with post-ligation cleanups performed as per protocol. Library amplification was carried out for 10 cycles, followed by cleanup using a 0.8× bead-based method. Library size was assessed using a high-sensitivity DNA bioanalyzer (Agilent Technologies), and library concentration was measured using a DeNovix double-stranded DNA high-sensitivity assay. Sequencing was conducted on an Illumina NextSeq 2000 instrument, generating 59-bp paired-end reads.

RNA-seq reads underwent low-quality filtering and adapter trimming using Trim Galore! v0.4.5, which incorporates Cutadapt v1.18 and FastQC v0.11.8 tools. Alignment to the human reference genome (GRCh38.95, Ensembl) was conducted using Star v2.7.5a. HTSeq (HTSeq-count tool v0.11.0) with default parameters and a GTF file containing GRCh38.95 annotation (Ensembl) determined the number of reads mapped to features.^36^ MultiQC assessed quality across all samples. DESeq2 v1.22.0 in R v3.5.3 conducted differential gene expression analysis, requiring a minimum of 5 reads per sample per gene. Pathway and GO term enrichment were analyzed using EnrichR v3.2 on genes with adjusted *p*-values <0.05. Further pathway analysis utilized the WikiPathway 2019 database.

### Data Analysis

The apparent permeability (*P*_app_) was calculated following eq 1, where d*Q*/d*t* is the rate of drug transport from one-half chamber to the other (either A-to-B or B-to-A), *A* is the exposed area of the tissue, and *C*_0_ is the initial concentration of the compound investigated in the donor compartment.
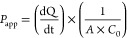
1

Efflux ratios (ERs) were determined according to eq 2 for each tissue. An ER ≥2 indicates active efflux, where 1–2 is less determinate.^37^
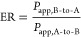
2

### Statistical Analysis

**Figure 1:**
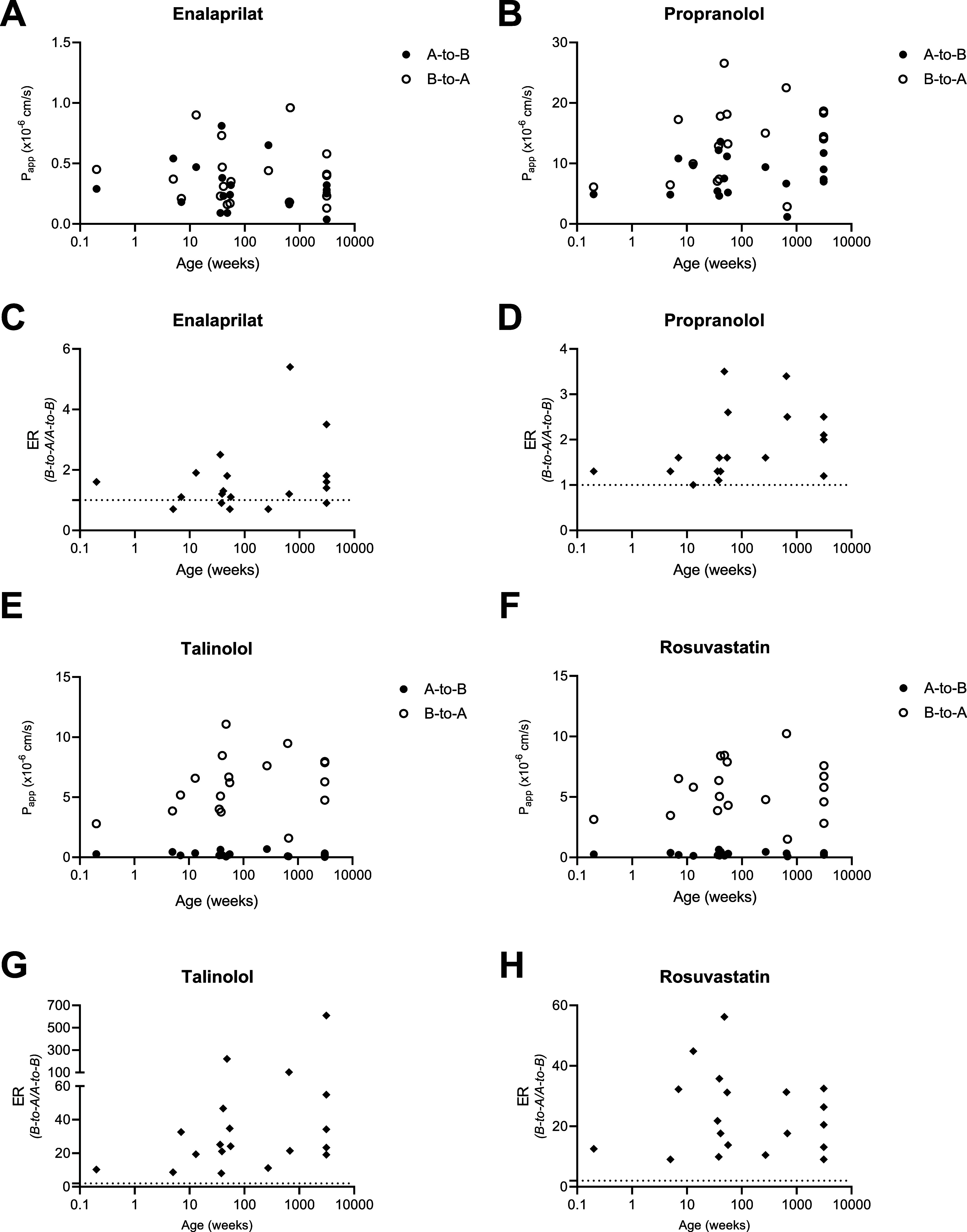
Apparent permeability (*P*_app_) and ER determined in enteroid monolayers. A-to-B: apical to basolateral transport. B-to-A: basolateral to apical transport. Succeeded experiments per substrate are described by *n* = 14 pediatric and 5 adult donor succeeded experiments. (A) *P*_app_ enalaprilat (paracellular transport). (B) *P*_app_ propranolol (transcellular transport). (C) ER enalaprilat with age. (D) ER propranolol with age. (E) *P*_app_ talinolol (P-gp transport). (F) *P*_app_ rosuvastatin (BCRP transport). (G) ER talinolol with age. (H) ER rosuvastatin with age. Bidirectional drug transport in human intestinal tissues.

**Figure 2:**
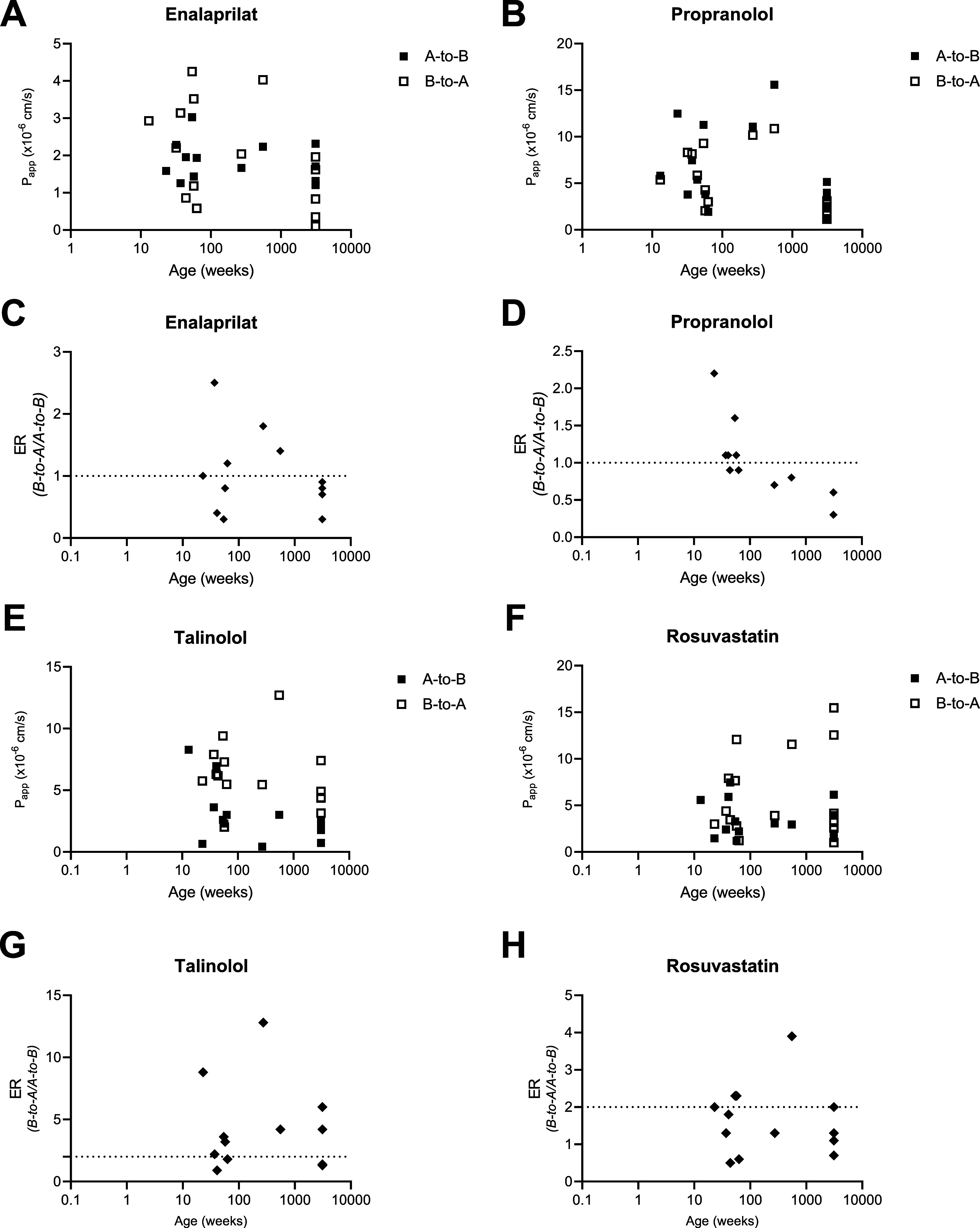
Apparent permeability (*P*_app_) and ER determined in tissue with the Ussing chamber. A-to-B: apical to basolateral transport. B-to-A: basolateral to apical transport. Succeeded experiments per substrate are described by *n* = A-to-B/B-to-A. (A) *P*_app_ enalaprilat (paracellular transport) *n* = 9/10 pediatrics, *n* = 4/5 adults. (B) *P*_app_ propranolol (transcellular transport) *n* = 10 pediatrics, *n* = 5 adults. (C) ER enalaprilat with age, *n* = 8 pediatric, *n* = 4 adults. (D) ER propranolol with age, *n* = 9 pediatric, *n* = 4 adults. (E) *P*_app_ talinolol (P-gp transport) *n* = 9/10 pediatrics, *n* = 4/6 adults. (F) *P*_app_ rosuvastatin (BCRP transport) *n* = 10/10 pediatrics, *n* = 4/6 adults. (G) ER talinolol with age, *n* = 8 pediatric, *n* = 4 adults. (H) ER rosuvastatin with age, *n* = 9 pediatric, *n* = 4 adults.

Data per patient are presented as the mean per experimental condition. *P*_app_ values in both transport directions (A-to-B versus B-to-A) were compared with a Wilcoxon paired signed rank test (Figures 1 and 2). A Spearman correlation was performed to explore a potential relation between *P*_app_ and age for Figures 1, 2, and 6. Kruskall–Wallis tests were used to compare the *P*_app_ values between enteroid monolayers and tissues. Significance was reported when *p* < 0.05. Statistical analyses were performed using GraphPad Prism 8.0.1.

## Results

### Tissue Collection

Ileum tissue was collected from 35 donors (pediatric and adult). The 28 pediatric tissues were obtained from continuity recovery, stoma construction, stoma revision, stoma closure, inguinal hernia correction, or sigmoid resection. From the 7 adult samples available from hemicolectomy surgery, 6 resulted in successful Ussing chamber experiments and 5 resulted in functional enteroid monolayers.

### Bidirectional Drug Transport in Enteroid Monolayers

For 14 pediatric donors, we cultured enteroid monolayers [median (range) age: 44 weeks (2 days–13 years)]. Accurate barrier formation was confirmed by FD4 apparent permeability (*P*_app_) in luminal to basolateral direction (A-to-B) and TEER (Supporting Information Figure 1).

Paracellular transport of enalaprilat in enteroid monolayers was similar in both transport directions, without an age-dependent trend (Figure 1B). For propranolol, B-to-A transport was higher than A-to-B transport, also reflected by the ER > 2 for 7 out of 19 donors (range ER_propranolol_: 1–4). Propranolol ER increased with age (Figure 1B,D). Mean ± SD *P*_app_ and ER values per donor can be found in Supporting Information Table 3. All statistical results can be found in Supporting Information Tables 5–7.

Transport (*P*_app_) of the substrates talinolol and rosuvastatin was higher in B-to-A in comparison to the A-to-B direction, resulting in ERs well above 2 (range ER_talinolol_: 8–223, range ER_rosuvastatin_: 9–45, Figure 1E–H, Supporting Information Table 3). This indicates the active efflux transport by P-gp and BCRP in the enteroid monolayers. No relation with age was observed for both transport directions, although the ER of talinolol showed a tendency to increase with age (Supporting Information Table 5).

Of the 28 pediatric tissues obtained, 11 were large and viable enough for an Ussing chamber drug transport experiment [median (range) age: 54 weeks (15 weeks −10 years)]. Figure 2A–D shows the *P*_app_ and ER of enalaprilat and propranolol in tissue with age. In line with physiochemical characteristics of these passive transport markers, A-to-B transport was comparable to B-to-A transport between donors for enalaprilat and propranolol, leading to ERs ≤2 for all donors except one (range ER_enalaprilat_: 0.3–2; range ER_propranolol_: 0.6–2). B-to-A transport decreased with age for both enalaprilat and propranolol, which was reflected in a concomitant decrease in ER. Variability between donors tended to be higher in pediatric patients than in adults for both enalaprilat and propranolol transport (*P*_app_). Exact *P*_app_ values and ER in the pediatric and adult donor groups are shown in Supporting Information Table 4. *P*-values for all age-related correlations can be found in Supporting Information Table 5. All statistical results can be found in Supporting Information Tables 5–7.

Figure 2E–H shows talinolol and rosuvastatin *P*_app_ and ER across intestinal tissue with age. As hypothesized, since P-gp and BCRP are both efflux transporters, the *P*_app_ of talinolol and rosuvastatin was higher in the B-to-A direction as compared to the A-to-B direction (Figure 2C,D), indicating efflux transport of both substrates. No age-related trend for *P*_app_ was observed for talinolol or rosuvastatin in both transport directions (Supporting Information Table 5).

### Comparison of Tissue versus Enteroids

Compared to tissue, enalaprilat *P*_app_ values were lower and B-to-A propranolol *P*_app_ values were higher in enteroid monolayers, indicating barrier differences between monolayers and tissue (Figures 1 and 2, Supporting Information Tables 3 and 4). This was also reflected in TEER, which was higher in enteroid monolayers compared to tissue (Supporting Information Figure 1). Interestingly, less variation in the pediatric age range was observed in enteroid monolayers compared to tissue (Figures 1A and 2A). Decreasing *P*_app_ and ER values with age were found for propranolol in tissue; however, in enteroid monolayers, an increase of propranolol ER was found with age.

Compared to tissue, A-to-B *P*_app_ values of talinolol and rosuvastatin were lower in enteroid monolayers, while B-to-A *P*_app_ values are in a similar range. This resulted in a higher ER in enteroid monolayers compared to tissues (Figures 1 and 2). In both tissue and enteroids, an age-related effect was not observed.

### Gene Expression

To gain more insights into the representation of enteroid monolayers compared to intestinal tissue, we performed bulk RNA sequencing analysis. Twenty-seven pediatric and 5 adult tissues and 13 pediatric and 5 adult enteroid monolayers were processed for RNA quantification. Principle component analysis (PCA) with normalized counts from the RNA-seq data showed a clear separation between the tissue and enteroid monolayer samples at PC1 (89%) (Supporting Information Figure 2). The difference in cell composition of whole tissue versus cell monolayer (epithelium only) may indicate functional changes between the tissue and enteroids.

**Figure 3:**
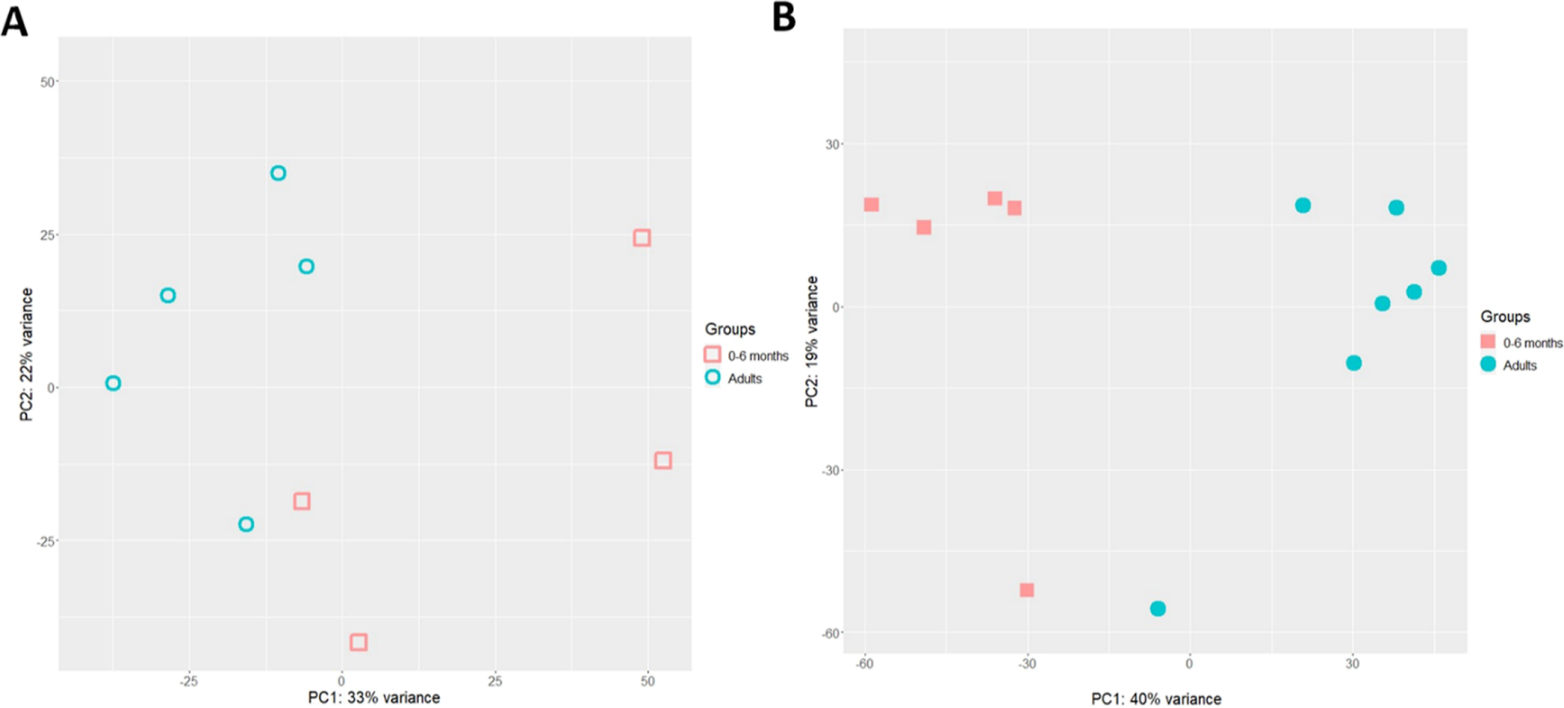
PCA of 0–6 month old donors and adults. (A) PCA of enteroid monolayers, red open squares: 0–6 month old enteroids *n* = 4, blue open circles: adult enteroids *n* = 5. (B) PCA of tissue, red filled squares: 0–6 month old tissue *n* = 5, blue filled circles: adult tissue donors *n* = 7.

**Figure 4:**
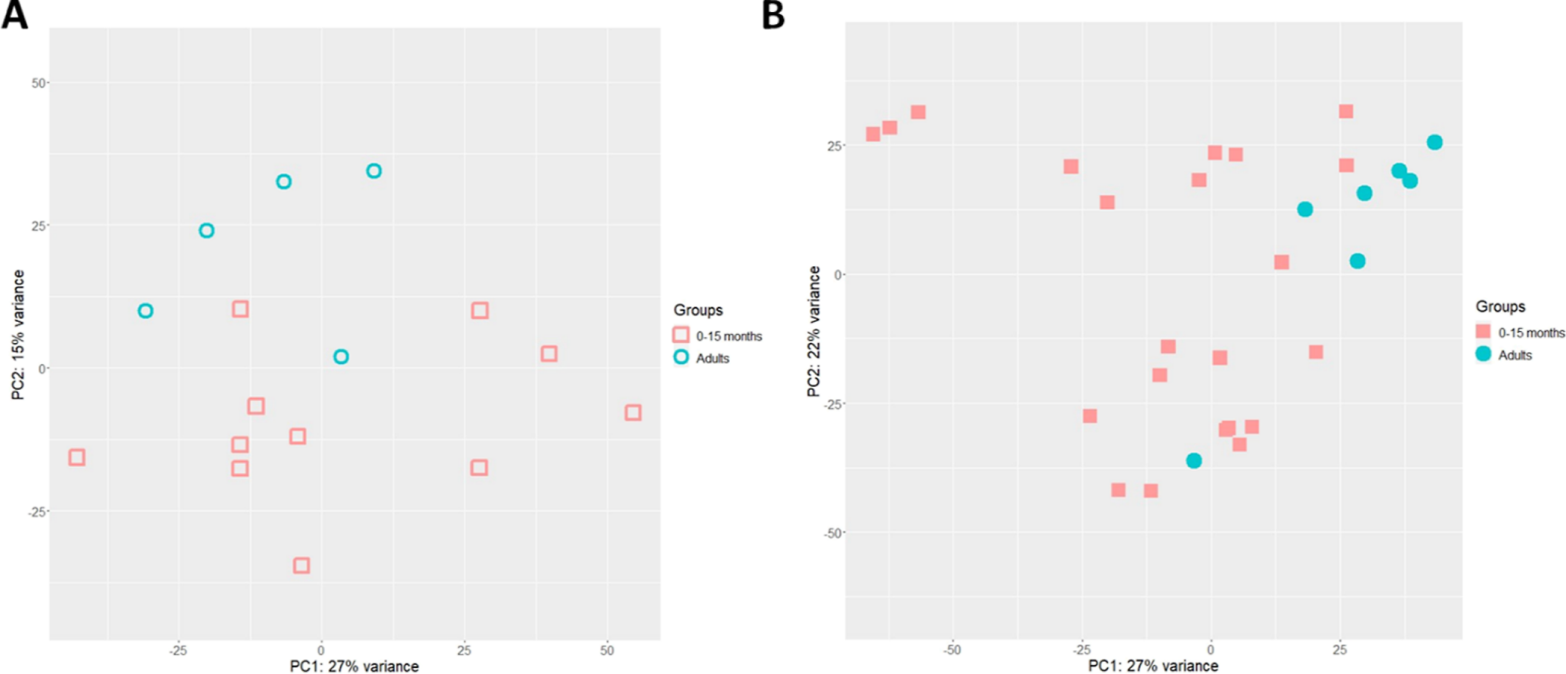
PCA of 0–15 month old donors and adults. (A) PCA of enteroid monolayers, red open squares: 0–15 month old enteroids *n* = 11, blue open circles: adult enteroids *n* = 5. (B) PCA of tissue, red filled squares: 0–15 month old tissue *n* = 22, blue filled circles: adult tissue donors *n* = 7.

**Figure 5:**
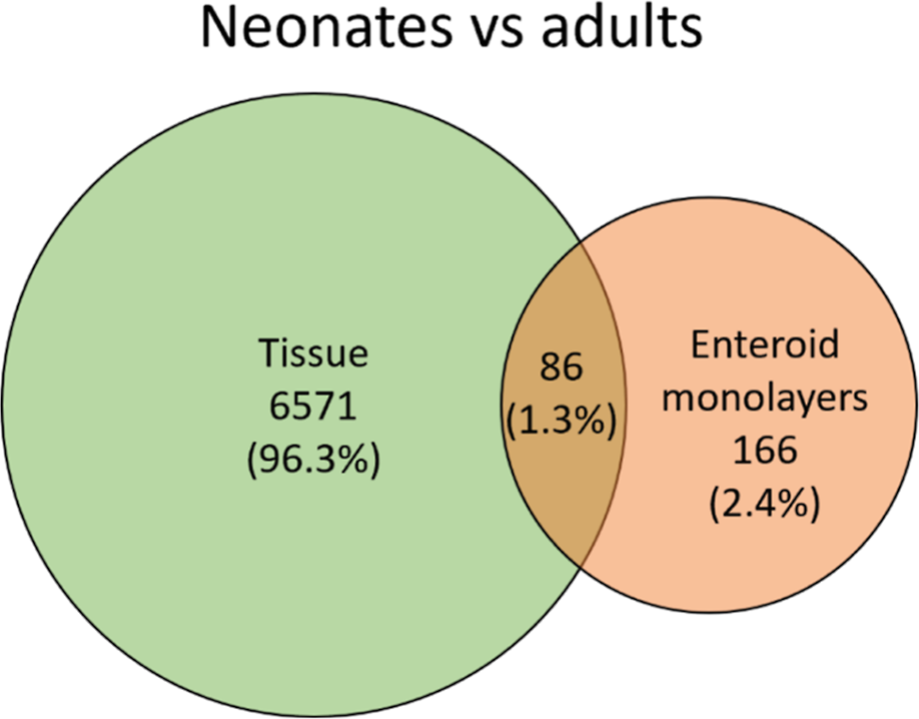
Venn diagram showing more DEGs in tissue compared to enteroid monolayers in neonates (*n*_tissue_ = 5, *n*_enteroids_ = 4) versus adults (*n*_tissue_ = 7, *n*_enteroids_ = 5).

For both enteroid monolayers as well as tissue, we compared the 0–6 month age group (neonates, *n*_tissue_ = 5, *n*_enteroids_ = 4) with the adult age group (*n*_tissue_ = 7, *n*_enteroids_ = 5). The 0–6 month age group because most PK changes are expected during the first months of life.^38^ The PCA for neonates versus adults showed a distinction of the two groups both in enteroid monolayers (PC1: 33%) as well as in tissue (PC1: 40%) (Figure 3). The PCA with tissue of 0–15 months to adult donors already shows that pediatric samples cluster more closely to the adults, indicating possible aging of the donors toward the adult genotype (Figure 4). More differential expressed genes (DEGs) were identified by comparing neonates with adults in tissue than in enteroid monolayers (Figure 5). In neonates versus adults 1.3% of DEGs overlapped between tissue and enteroid monolayers.

**Figure 6:**
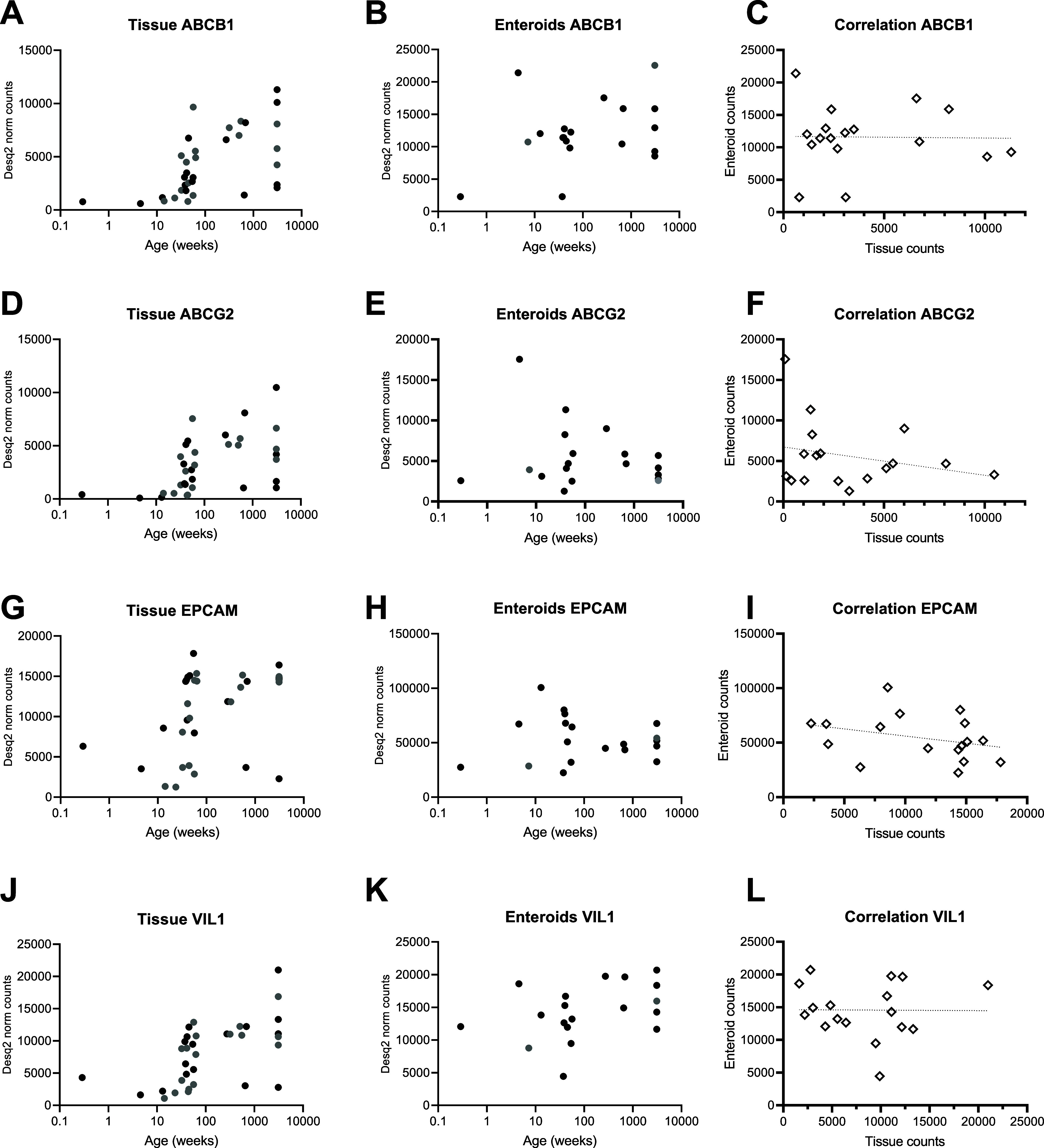
Gene expression of ABCB1 (P-gp) and ABCG2 (BCRP) in tissue *n* = 34 and in enteroid monolayers *n* = 18 across the age range. Black dots are donors with tissue and enteroid measurement. Gray dots miss one of the two models. (A) ABCB1 in tissue, (B) ABCB1 in enteroid monolayers, (C) correlation of ABCB1 expression between tissue and enteroid monolayers, (D) ABCG2 in tissue, (E) ABCG2 in enteroid monolayers, (F) correlation of ABCG2 expression between tissue and enteroid monolayers, (G) EPCAM in tissue, (H) EPCAM in enteroid monolayers, (I) correlation of EPCAM expression between tissue and enteroid monolayers, (J) VIL1 in tissue, (K) VIL1 in enteroid monolayers, and (L) correlation of VIL1 between tissue and enteroid monolayers.

Next, we focused on *ABCB1* (P-gp) and *ABCG2* (BCRP), the efflux transporters investigated for functionality in Figures 1 and 2. Both genes were expressed in all samples, but their expression was highly variable in both enteroids and tissue (Figure 6A–F). Enteroid monolayer and tissue expressions of *ABCB1* and *ABCG2* did not correlate (Figure 6C,F). In tissue, *ABCB1* and *ABCG2* expression was higher with age, r:0.64; *p* < 0.0001 and *r*: 0.58; *p* = 0.003, respectively (Figure 6A,D). In enteroid monolayers, no relation with age was observed for *ABCB1* and *ABCG2* expression (Figure 6B,E). Interestingly, in tissue, we also found an age-related effect for the epithelial cell marker EPCAM and enterocyte marker villin-1 (VIL1) (Figure 6G–L). This might indicate lower epithelial cell input in the RNA tissue samples of the younger donors. Statistical Spearman *r*- and *p*-values can be found in Supporting Information Table 8.

**Figure 7:**
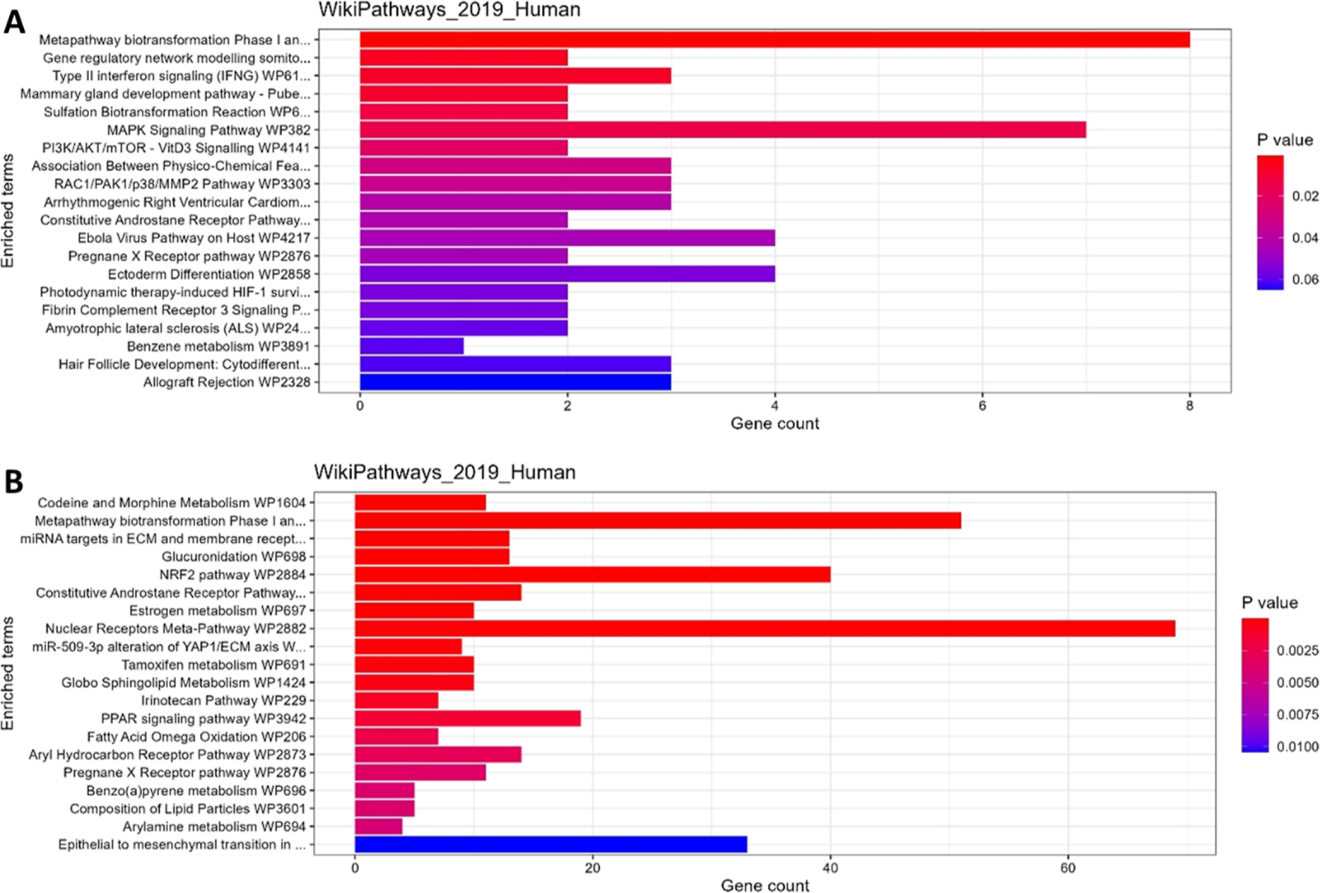
Top 20 pathways from WikiPathways (EnrichR) that were significantly enriched between 0 and 6 month old and adult enteroid monolayers (A) and tissue (B).

Lastly, unsupervised pathway analysis was performed to determine enriched pathway differences between neonates and adults in enteroid monolayers and tissue. The pathway analysis showed significant differences in several PK-related pathways in both enteroids and tissue (Figure 7). The top 20 significantly different pathways between neonates and adults included metapathway biotransformation phase 1 and 2 metabolism, CAR and PXR pathway in both enteroid monolayers as well as tissue. These results indicate that these age-related pathway differences might be preserved in the enteroids. In tissue, more PK-related pathways were identified, as glucuronidation and AhR pathway.

## Discussion

We successfully used enteroids to study passive and active drug transport from neonates to adults. Moreover, enteroid transport functionality is largely in line with transport functionality using ex vivo tissue in Ussing experiments. Our explorative RNA sequencing data show age-related variation in drug metabolism genes between neonates and adults in both tissue and enteroids. But overall, enteroids show less age-related variation in gene expression than tissue.

Successful bidirectional drug transport enteroid monolayer experiments were performed for 14 pediatric donors (9 donors <1 year of age), as confirmed by TEER, FD4, and passive transport markers enalaprilat and propranolol. Of the 28 pediatric tissues (5 donors <1 year of age), we successfully performed 11 Ussing chamber experiments. We were able to perform more intestinal enteroid than Ussing experiments in children <1 year of age, as less tissue is needed to culture organoids than for Ussing experiments.

Interestingly, while most *P*_app_ and ER values were quite similar between enteroids and tissue, apical to basolateral transport was much lower, even close to absent in enteroids rather than tissue for paracellular and efflux transport substrates. In contrast, transcellular transport was higher in B-to-A direction in enteroids. Both are most likely caused by differences in physiological barrier: a tight monolayer of cells caused by the presence of tight junctions and multicellular tissue. The presence of a tighter barrier in enteroid monolayers was also confirmed by higher TEER measurements in enteroids compared to tissue and is similar to drug transport studies with other cell lines.^39−41^ Additionally, we had to use low (70) rpm during enteroid transport studies due to observed barrier disruption when using a higher rpm speed. This might not have been optimal for transcellular permeability. Variability of the passive transport markers was higher in pediatric tissue compared to that in pediatric enteroids. This might indicate that upon enteroid culturing, individual differences in barrier integrity disappear, as upon in vitro culture, the individual in vivo environment decreases.

We used RNA sequencing to further compare enteroids with tissue and explore age-related variation between enteroids and tissue. Unsupervised clustering showed that tissue and enteroid monolayers are distinct from each other (89%). Therefore, follow-up RNA-seq analysis was performed within the tissue and enteroid monolayer groups separately because of input differences. Unsupervised clustering within the two models showed that neonates and adults clustered separately from each other in tissue; in organoids, these clusters were still present but less profound. The number of differentially expressed genes in neonates versus adults decreased upon enteroid culturing compared to tissue. This is not unexpected, as upon in vitro culturing, the intestinal microenvironment (lamina propria) changes and systemic factors as diet and microbiome disappear.^42^

Additionally, we highlighted gene expression of *ABCB1* (P-gp) and *ABCG2* (BCRP) as they are involved in the DT discussed in this study. In tissue, a trend of increasing expression with age for both genes was observed, which was not reflected in enteroid monolayers (Figure 6). The trend observed in tissue could not be confirmed on a functional level in tissue due to the low number of inclusions in the youngest age range. In enteroids, a weak trend was observed for an increased ER of talinolol (P-gp substrate) with age. Interestingly, to explore this difference in age-related variation between tissue and organoids, expression of epithelial cell marker EPCAM and enterocyte marker VIL-1 also showed an age-related trend. As P-gp and BCRP2 are only expressed in epithelial cells expressing EPCAM and VIL-1, this might indicate that the effect found is caused by differences in cell type composition in tissue specimens used for RNA isolation instead of in their age (Figure 6). Unsupervised pathway analysis showed that several PK-related pathways were significantly differed between neonates and adult donors, in both tissue and in enteroids. This is in line with other studies showing age-related variation in expression of drug metabolizing enzymes in intestinal tissue^7,43−45^ and is encouraging for the use of pediatric enteroids to study age-related variation in intestinal drug disposition.

In a previous RNA-seq study with young adult mice (10–15 weeks old) and old adult mice (85–115 weeks old) intestinal tissue crypts and 3D enteroids, DEGs in young versus old adult enteroids were drastically lower than in tissue crypts, where more DEGs were found between young and old adult mice.^42^ The authors hypothesize that aging phenotypes of the intestine in enteroids change due to the in vitro absence of cell extrinsic factors of its microenvironment in vivo. Differences between the three small intestinal regions, which are more dependent on cell intrinsic factors, were more defined in intestinal enteroids.^42^ The microenvironment of the intestine changes drastically in the first months of life, which at this moment is not considered in organoid culturing. For instance, cesarean section versus natural birth, exposure to bacteria, shift of food intake, antibiotic use are all factors influencing the intestinal microenvironment during the first period of life.^8,46^ Together with our data, these data strongly suggest that optimization of the culture conditions, such as addition of growth factors or bacterial metabolites, could provide more similar conditions to the in vivo situation for both neonatal as for adult enteroids.^42,47^

Adeniyi-Ipadeola et al. in 2023 and Noel et al. in 2021 reported differences between pediatric and adult enteroid monolayers, which we could not confirm. Enteroid monolayer TEER was lower, as well as enterocyte cell height in infant (10–22 weeks *n* = 3) and in 2–5 year old enteroid monolayers (*n* = 2) compared to adult enteroid monolayers. Additionally, CLDN2 expression in pediatric enteroid monolayers is higher than adult, suggesting possible increased leakiness in intestines from children.^15,48^ Importantly, a comparison with adult tissue is missing. In this study, TEER did not differ between age groups in tissue nor in enteroid monolayers (Supporting Information Figure 1). Although CLDN2 expression was higher in pediatric tissues (6–15 months) than adult tissue (*p* = 0.001), this was not the case in enteroid monolayers (*p* > 0.999) (Supporting Information Figure 3).

In the literature, information on intestinal drug transport in pediatrics is scarce. On a functional level, there is our previous proof of concept publication on the Ussing chamber for pediatric samples.^16^ Here, we did not find a relationship with age on a continuous age scale for P-gp and BCRP [median (range) age: 44 weeks (8 weeks–17 years)]. In fresh intestinal tissue, BCRP, P-gp, and PEPT1 protein abundance were lower in 0–2 year olds compared to adults.^7^ However, the adult age group was small and exhibited high variability between samples. The study conducted by Goelen et al.^44^ in 2023 showed that drug transporters were stable in 2–15 year olds, as were most DMEs; only CYP3A4 and CES2 abundance increased with age. Unfortunately, the younger age group was missing from this cohort (8). It is within the youngest specific age groups (0–1 year) where variations in ADME genes are expected.^38^ Previously, our lab showed proof of concept to study pediatric drug transport with the Ussing chamber; here, we did not find age-related differences within the pediatric age range with similar drug substrates [median (range) age: 44 weeks, 8 weeks-17 years, *n* = 15].

This study has some limitations. Although the Ussing chamber experimental setup comes close to the in vivo situation, it still has its challenges. For the youngest donors (<1 year old), Ussing chamber experiments were hampered by the limited availability of tissue and, when available, its limited size. Especially for the children <6 months, left-over tissue samples are very small to preserve as much as possible viable intestine for the patient. The small size prevents Ussing experiments, but it is possible to isolate enteroids from tiny pieces of tissue. Next to that, in this study, bulk RNA-seq data was derived; single-cell RNA-seq could provide more accurate information in expression differences between the different age groups and the composition of intestinal cell types, which might influence drug absorption.^42^ However, it should be taken into account that RNA expression does not always translate well to protein abundance and functionality.^49−51^ Despite our findings that we found a correlation with age for some transporters, a larger data set may be needed to unequivocally show the age-related variation (or lack thereof) in transporter activity. Other factors, explaining the observed variability, cannot be ruled out, and confirmation of our findings should be a focus on future studies.

To summarize, this study provides the first data to further develop pediatric enteroids to study age-related variation in drug transport and metabolism. Enteroid transport functionality was overall in line with Ussing chamber to obtain data. Additionally, pathway analysis showed similar PK-related differences between neonates and adults in both tissue and enteroid monolayers. Given the challenge to elucidate the effect of developmental changes in the pediatric age range in vivo, intestinal enteroids derived from pediatric patients might provide a versatile experimental platform to study pediatric phenotypes; however, a lot of questions remain to be answered.

## Supporting Information Available

Compositions of buffers and media used in enteroid culture, donor characteristics, statistics, barrier integrity, apparent permeability values per donor, and additional RNA-seq PCA and gene expression (PDF)
