# Supplementary material for: Enteroids to Study Pediatric Intestinal Drug Transport
Source: Mol Pharm. 2024 Sep 16;21(10):4983–94. doi: 10.1021/acs.molpharmaceut.4c00339 (PMC11462498; doi:10.1021/acs.molpharmaceut.4c00339)
Supplement: Supplementary file 1 — mp4c00339_si_001.pdf [file mp4c00339_si_001.pdf]

Supplementary

Enteroids to study pediatric intestinal drug transport

Eva J. Streekstra, Marit Keuper-Navis, Jeroen J.M.W. van den Heuvel, Petra van den Broek, Martijn W.J. Stommel, Willem de Boode, Sanne Botden, Sander Bervoets, Luke O'Gorman, Rick Greupink, Frans G. M. Russel, Evita van de Steeg, Saskia N. de Wildt

Supporting information: additional experimental details material and methods, including donor characteristics, statistics, barrier integrity, apparent permeability values per donor and additional RNAseq PCA and gene expression.

Supplementary Table 1: compositions of buffers and media used in enteroid culture

Solution	Components	End concentration	Supplier
**Storage Buffer**	Williams' E		Gibco, Thermo Fisher Scientific, Walthman, MA
	Glucose	2.8 mg/mL	Sigma-Aldrich, Burlington, MA
	Amphoterictin B	2.5 µg/mL	Gibco, Thermo Fisher Scientific, Walthman, MA
	Gentamicin	500 µg/mL	Gibco, Thermo Fisher Scientific, Walthman, MA
	GlutaMAX	2mM	Gibco, Thermo Fisher Scientific, Walthman, MA
	HEPES	10mM	Gibco, Thermo Fisher Scientific, Walthman, MA
**Wash Buffer**	PBS		Gibco, Thermo Fisher Scientific, Walthman, MA
	Pen/Strep	100 U/mL	Gibco, Thermo Fisher Scientific, Walthman, MA
	Gentamicin	500 µg/mL	Gibco, Thermo Fisher Scientific, Walthman, MA
	Amphotericin B	2,5 µg/mL	Gibco, Thermo Fisher Scientific, Walthman, MA
	Primocin	50 µg/mL	Invitrogen, Carlsbad, CA
**Crypt releasing solution**	Wash buffer		See above
	EDTA	4 mM	Sigma-Aldrich, Burlington, MA
	DTT	2 mM	Roche, Basel, Switserland
**Advance DMEM +++**	Advanced DMEM		Gibco, Thermo Fisher Scientific, Walthman, MA
	Pen/strep	100 U/mL	Gibco, Thermo Fisher Scientific, Walthman, MA
	Glutamax	2 mM	Gibco, Thermo Fisher Scientific, Walthman, MA
	HEPES	10 mM	Gibco, Thermo Fisher Scientific, Walthman, MA
**Organoid Growth Medium**	IntestiCult Organoid Growth Medium (Human)		STEMCELL™ Technologies, Vancouver, BC, Canada
	IntestiCult Supplements		STEMCELL™ Technologies, Vancouver, BC, Canada
	Pen/Strep	100 U/mL	Gibco, Thermo Fisher Scientific, Walthman, MA
	Rock-inhibitor (Y-27632)*	10 µM	STEMCELL™ Technologies, Vancouver, BC, Canada
**Organoid Differentiation Medium**	IntestiCult Organoid Differentiation Medium (Human)		STEMCELL™ Technologies, Vancouver, BC, Canada
	IntestiCult Organoid Supplements		STEMCELL™ Technologies, Vancouver, BC, Canada
	Pen/Strep	100 U/mL	Gibco, Thermo Fisher Scientific, Walthman, MA
	DAPT	5 µM	STEMCELL™ Technologies, Vancouver, BC, Canada
**Coating solution**	Acetic acid	0.1%	Sigma-Aldrich, Burlington, MA
	Rat tail Collagen Type 1	100 µg/mL	Ibidi, Fitchburg, WI
**Transport buffer**	HBSS		Sigma-Aldrich, Burlington, MA
	HEPES	10 mM	Gibco, Thermo Fisher Scientific, Walthman, MA
	Glutamax	2 mM	Gibco, Thermo Fisher Scientific, Walthman, MA

*addition of rock-inhibitor the first 3 days after isolation, monolayer seeding or thawing of enteroids

*Supplementary Table 2: donor characteristics. * & **: same donor included at different age points.*

Age (weeks)	Intestinal Region	Gestational age	(Previous) disease state	Type of surgery	Gender	Transport experiment	RNA sequencing
0.29	Ileum	34+6	Ileum volvulus (obstruction)	Continuity recovery	Female	Enteroids	Tissue + Enteroids
4.57	Mid Ileum	34+5	Previous NEC perforation	Laparotomy to correct inguinal hernia	Male	Enteroids	Tissue + Enteroids
7.29	Distal ileum (50cm voor colon)	Term	Colon Hirschsprung	Intestinal resection. Stoma construction	Male	Enteroids	Enteroids
13.00	Terminal ileum	31+0	Previous NEC 2B	Stoma closure surgery	Male	Enteroids	Tissue + Enteroids
14.14	Ileum	25+2	Previous NEC 3B	Stoma closure surgery	Male	-	Tissue
15.00	Ileum	unknown	Colon Hirschsprung	Stoma closure surgery	Male	Tissue	-
23.43	Terminal ileum	39+5	Meconium peritonitis. Colon Hirschsprung	Stoma closure surgery	Female	Tissue	Tissue
32.14	Ileum	Term 40	Meckel's diverticulum	Small intestine resection	Male	Tissue	Tissue
32.29*	Terminal ileum	37+2	Previous atypical NEC	Ileostomy revision	Male	-	Tissue
37.42	Ileum	29+1	Meconium plug	Stoma closure surgery	Male	Tissue	Tissue + Enteroids
38.57	Terminal ileum	39+0	Hirschsprung	Stoma closure surgery	Female	Enteroids	Tissue + Enteroids
40.29	Ileum	unknown	Hirschsprung	Stoma closure surgery	Male	Enteroids	Tissue + Enteroids
40.85**	Terminal ileum	32+1	Previous NEC	Stoma closure surgery	Female	Tissue	Tissue
41.43*	Ileum	37+2	Previous atypical NEC, colon ischemia	Stoma closure surgery	Male	Tissue + Enteroids	Tissue + Enteroids
43.86	Ileum	32+2	Previous NEC 2B	Stoma closure surgery	Male	-	Tissue
45.14**	Ileum	32+1	Previous NEC	Ileostomy revision	Female	-	Tissue
45.29	Ileum	25+0	Previous NEC. Short bowel syndrome	Stoma closure surgery	Male	Enteroids	Tissue + Enteroids
54.14	Terminal ileum	31+5	Previous NEC 3B	Stoma closure surgery	Male	Enteroids	Tissue + Enteroids
56.29	Ileum	32+2	Previous NEC 3B	Stoma closure surgery	Female	Tissue + Enteroids	Tissue + Enteroids
56.57	Terminal Ileum	25+0	Meconium plug	Stoma closure surgery	Female	Tissue	Tissue + Enteroids
56.86	Ileum	unknown	Mesenterial cyst	Stoma closure surgery	Female	-	Tissue + Enteroids
62.71	Ileum	Term 40+6	Distal ileum atresia. Hirschsprung	Stoma closure surgery	Female	Tissue	Tissue
63.86**	Ileum	32+1	Previous NEC	Ileostomy revision	Female	-	Tissue

273.57	Terminal ileum	unknown	Colon Hirschsprung	Stoma closure surgery	Male	Tissue + Enteroids	Tissue + Enteroids
318.00	Terminal ileum	unknown	Colon Hirschsprung	Stoma closure surgery	Male	-	Tissue
506.57	Terminal ileum	37+4	Gastroschisis	Stoma closure surgery	Female	-	Tissue
554.86	Ileum	31+3	Obstipation caused by underlying genetic disease	Stoma construction	Female	Tissue	Tissue
648.00	Terminal ileum	unknown	Obstipation caused by underlying genetic disease	Sigmoid resection	Male	Enteroids	Tissue + Enteroids
687.86	Terminal ileum	unknown	Crohn's diseases	Stoma closure surgery	Female	Enteroids	Tissue + Enteroids
Adult 1	Terminal ileum	-	Colon carcinoma	(right-hemi) colectomy	-	Tissue	Tissue + Enteroids
Adult 2	Terminal ileum	-	Colon carcinoma	(right-hemi) colectomy	-	Tissue + Enteroids	Tissue + Enteroids
Adult 3	Terminal ileum	-	Colon carcinoma	(right-hemi) colectomy	-	Tissue	Enteroids
Adult 4	Terminal ileum	-	Colon carcinoma	(right-hemi) colectomy	-	Tissue	Tissue + Enteroids
Adult 5	Terminal ileum	-	Colon carcinoma	(right-hemi) colectomy	-	Tissue + Enteroids	Tissue + Enteroids
Adult6	Terminal ileum	-	Colon carcinoma	(right-hemi) colectomy	-	Enteroids	Tissue
Adult 7	Terminal ileum	-	Colon carcinoma	(right-hemi) colectomy	-	Enteroids	-
Adult 8	Terminal ileum	-	Colon carcinoma	(right-hemi) colectomy	-	Enteroids	Tissue + Enteroids

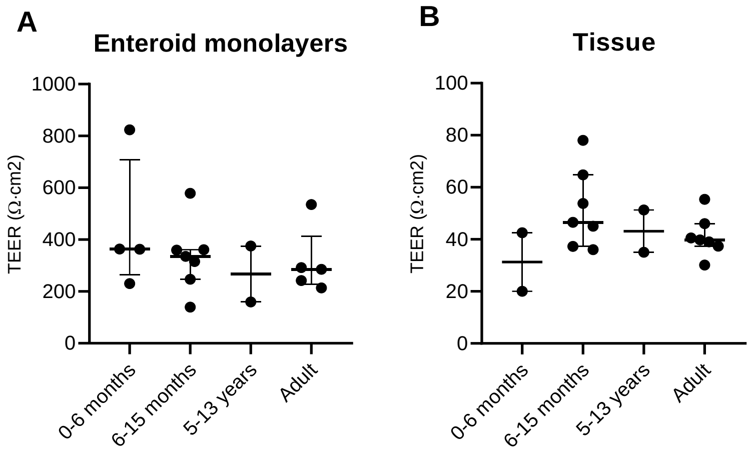

Supplementary Figure 1 Trans epithelial electrical resistance (TEER) in enteroid monolayers (A) and tissue explants in Ussing chamber (B). Data represented in median ± IQR.

Supplementary Table 3: apparent permeability values (mean ± SD) and efflux ratios per donor in enteroid monolayers

Age (weeks)		Paracellular – enalaprilat	Transcellular – propranolol	P-gp – talinolol	BCRP – rosuvastatin
**0.29**	A-to-B	0.3 ± 0.2 (3)	5 ± 0.3 (3)	0.3 ± 0.1 (3)	0.3 ± 0.01 (3)
	B-to-A	0.5 ± 0.03 (3)	6 ± 0.3 (3)	3 ± 0.1 (3)	3 ± 0.2 (3)
	ER	2	1	10	13
**4.57**	A-to-B	0.5 ± 0.2 (3)	5 ± 0.5 (3)	0.4 ± 0.2 (3)	0.4 ± 0.1 (3)
	B-to-A	0.4 ± 0.1 (3)	7 ± 0.4 (3)	4 ± 0.1 (3)	3 ± 0.1 (3)
	ER	0.7	1	9	9
**7.29**	A-to-B	0.2 ± 0.1 (3)	11 ± 0.8 (3)	0.2 ± 0.1 (3)	0.2 ± 0.1 (3)
	B-to-A	0.2 ± 0.01 (3)	17 ± 0.5 (3)	5 ± 0.4 (3)	7 ± 0.5 (3)
	ER	1	2	33	32
**13**	A-to-B	0.5 ± 0.1 (3)	10 ± 1.0 (3)	0.3 ± 0.1 (3)	0.1 ± 0.1 (3)
	B-to-A	0.9 ± 0.2 (3)	10 ± 1.1 (3)	7 ± 0.8 (3)	6 ± 0.7 (3)
	ER	2	1	19	45
**37.2**	A-to-B	0.1 ± 0.1 (3)	5 ± 0.2 (3)	0.2 ± 0.03 (3)	0.2 ± 0.1 (3)
	B-to-A	0.2 ± 0.02 (3)	7 ± 0.5 (3)	4 ± 0.2 (3)	4 ± 0.1 (3)
	ER	3	1	25	22
**38.6**	A-to-B	0.8 ± 0.1 (3)	12 ± 1 (3)	0.6 ± 0.1 (3)	0.6 ± 0.1 (3)
	B-to-A	0.7 ± 0.1 (3)	13 ± 0.6 (3)	5 ± 0.3 (3)	6 ± 0.6 (3)
	ER	0.9	1	8	10
**40.29**	A-to-B	0.4 ± 0.2 (3)	5 ± 0.1 (3)	0.2 ± 0.1 (3)	0.1 ± 0.1 (3)
	B-to-A	0.5 ± 0.1 (3)	7 ± 0.5 (3)	4 ± 0.2 (3)	5 ± 0.3 (3)
	ER	1	2	21	36
**41.4**	A-to-B	0.2 ± 0.03 (3)	14 ± 0.7 (3)	0.2 ± 0.0 (3)	0.5 ± 0.04 (3)
	B-to-A	0.3 ± 0.04 (3)	18 ± 2 (3)	9 ± 1 (3)	8 ± 1 (3)
	ER	1	1	47	18
**45.3**	A-to-B	0.1 ± 0.1 (3)	8 ± 0.6 (3)	0.1 ± 0.02 (3)	0.2 ± 0.02 (3)
	B-to-A	0.2 ± 0.01 (3)	27 ± 1 (3)	11 ± 0.3 (3)	8 ± 0.3 (3)
	ER	2	4	223	56
**54.14**	A-to-B	0.2 ± 0.1 (3)	11 ± 1 (3)	0.2 ± 0.2 (3)	0.3 ± 0.1 (3)
	B-to-A	0.2 ± 0 (3)	18 ± 1 (3)	7 ± 0.4 (3)	8 ± 0.6 (3)
	ER	0.7	2	35	31
**56.29**	A-to-B	0.3 ± 0.03 (3)	5 ± 0.3 (3)	0.3 ± 0.04 (3)	0.3 ± 0.02 (3)
	B-to-A	0.4 ± 0.1 (3)	13 ± 0.6 (3)	6 ± 0.3 (3)	4 ± 0.3 (3)
	ER	1	3	24	14
**273.57**	A-to-B	0.7 ± 0.2 (3)	9 ± 1 (3)	0.7 ± 0.2 (3)	0.5 ± 0.1 (3)
	B-to-A	0.4 ± 0.2 (3)	15 ± 1 (3)	8 ± 0.6 (3)	5 ± 0.4 (3)
	ER	0.7	2	11	11
**648**	A-to-B	0.2 ± 0.03 (2)	7 ± 0.9 (2)	0.1 ± 0.03 (2)	0.3 ± 0.03 (2)

	B-to-A	0.2 ± 0.01 (2)	23 ± 1 (2)	9 ± 0.1 (2)	10 ± 0.02 (2)
	ER	1	3	103	31
**687.86**	A-to-B	0.2 ± 0.1 (3)	1 ± 0.3 (3)	0.1 ± 0.03 (3)	0.1 ± 0.02 (3)
	B-to-A	1 ± 0.1 (3)	3 ± 0.2 (3)	2 ± 0.2 (3)	2 ± 0.2 (3)
	ER	5	2	21	18
**Adult 1**	A-to-B	0.04 ± 0.0 (2)	7 ± 0.02 (2)	0.04 ± 0.03 (2)	0.2 ± 0.03 (2)
	B-to-A	0.1 ± 0.1 (3)	18 ± 1.0 (3)	8 ± 0.4 (3)	7 ± 0.5 (3)
	ER	3	2	192	33
Adult 2	A-to-B	0.3 ± 0.1 (3)	7 ± 0.6 (3)	0.3 ± 0.1 (3)	0.3 ± 0.1 (3)
	B-to-A	0.6 ± 0.2 (3)	14 ± 1 (3)	8 ± 0.2 (3)	8 ± 0.6 (3)
	ER	2	2	19	9
**Adult 3**	A-to-B	0.3 ± 0.1 (3)	7 ± 1 (3)	0.1 ± 0.04 (3)	0.2 ± 0.1 (3)
	B-to-A	0.4 ± 0.1 (3)	14 ± 1 (3)	8 ± 0.2 (3)	8 ± 0.6 (3)
	ER	1	2	53	27
Adult 4	A-to-B	0.3 ± 0.3 (2)	9 ± 0.5 (2)	0.2 ± 0.2 (2)	0.4 ± 0.3 (2)
	B-to-A	0.2 ± 0.2 (3)	19 ± 0.3 (3)	8 ± 0.2 (3)	8 ± 0.6 (3)
	ER	1	2	34	20
Adult 5	A-to-B	0.3 ± 0.01 (3)	12 ± 4 (3)	0.2 ± 0.1 (3)	0.4 ± 0.1 (3)
	B-to-A	0.4 ± 0.1 (3)	14 ± 1 (3)	5 ± 0.4 (3)	5 ± 0.4 (3)
	ER	2	1	23	13

Supplementary Table 4: apparent permeability values (mean ± SD) and efflux ratios per donor in Ussing chamber

Age (weeks)		Paracellular – enalaprilat	Transcellular – propranolol	P-gp - talinolol	BCRP – rosuvastatin
**15.00**	A-to-B	2 (1)	12 (1)	8 (1)	5 (1)
	B-to-A				
	ER				
**23.43**	A-to-B	2 (1)	4 (1)	0.7 (1)	1 (1)
	B-to-A	2 (1)	8 (1)	6 (1)	3 (1)
	ER	1	2	9	2
**37.42**	A-to-B	2 ± 0.4 (2)	5 ± 2 2)	7 ± 5 (2)	2 ± 0.9 (2)
	B-to-A	0.9 ± 0.03(2)	6 ± 1 (2)	6 ± 1 (2)	4 ± 2 (2)
	ER	0.4	1	0.9	*2*
**40.85**	A-to-B	1 (1)	7 ± 3 (2)	4 ± 2 (2)	6 ± 4.3 (2)
	B-to-A	3 ± 0.7 (2)	8 ± 2 (2)	8 ± 1 (2)	8 ± 0.1 (2)
	ER	2	1	2	1
**41.43**	A-to-B		6 (1)		7 (1)
	B-to-A	3 (1)	5 (1)	6 (1)	3 (1)
	ER		0.9		0.5
**54.14**	A-to-B	3 (1)	11 (1)	3 (1)	3 (1)
	B-to-A	4 ± 0.9 (2)	9 ± 0.1 (2)	9 ± 0.4 (2)	8 ± 0.8 (2)
	ER	0.3	*2*	*13*	1
**56.29**	A-to-B	1 ± 0.7 (3)	4 ± 0.7 (3)	2 ± 0.7 (3)	1 ± 0.2 (3)
	B-to-A	1 (1)	4 (1)	7 (1)	3 (1)
	ER	0.8	1	3	2
**56.57**	A-to-B				
	B-to-A	4 (1)	2 (1)	2 (1)	12 (1)
	ER				
**62.71**	A-to-B	2 ± 0.5 (2)	11 ± 8 (2)	3 ± 0.7 (2)	2 ± 0.7 (2)
	B-to-A	2 (1)	10 (1)	5 (1)	1 (1)
	ER	1	0.9	2	0.6
**273.57**	A-to-B	2 (1)	2 ± 1 (2)	0.4 ± 0.2 (2)	3 (1)
	B-to-A	0.6 (1)	3 ± 2 (2)	5 ± 3 (2)	4 (1)
	ER	2	0.7	4	*4*
**554.86**	A-to-B	2 ± 0.4 (2)	16 ± 4 (2)	3 ± 0 (2)	3 (1)
	B-to-A	4 ± 0.03 (2)	11 ± 6 (2)	13 ± 5 (2)	12 ± 6 (2)
	ER	1	0.8	*4*	2
**Adult 2**	A-to-B				
	B-to-A		2 ± 0.1 (2)	5 ± 3 (2)	15 (1)
	ER				
**Adult 5**	A-to-B	1 ± 0.2 (3)	2 ± 1 (3)	2 ± 1.4 (3)	2 ± 0.4 (3)
	B-to-A	0.4 ± 0.1 (3)	2 ± 0.1 (3)	3 ± 0.3 (3)	1 ± 0.4 (3)
	ER	0.3	0.6	1	0.7
**Adult 6**	A-to-B	2 (1)	5 ± 2 (2)	2 ± 1 (2)	6 ± 1 (2)
	B-to-A	2 (1)	3 (1)	7 (1)	13 (1)
	ER	1	0.6	4	2
**Adult 7**	A-to-B	1 ± 0.01 (2)	3 ± 0.1 (2)	0.7 ± 0.2 (2)	2 ± 0.1 (2)

	B-to-A	0.8 ± 0.3 (3)	1 ± 0.3 (3)	4 ± 1.2 (3)	3 ± 1 (3)
	ER	0.7	0.3	6	1
Adult 8	A-to-B	2 ± 0.7 (3)	4 ± 3 (3)	2 ± 1 (3)	4 ± 1 (3)
	B-to-A	2 ± 2 (2)	2 ± 2 (2)	3 ± 3 (2)	4 ± 2 (2)
	ER	0.8	0.6	1	1

Supplementary Table 5: p-vales for Spearman correlation of apparent permeability with age of Figure 1 and 2.

		Enalaprilat		Propranolol		Talinolol		Rosuvastatin	
		r	p	r	p	r	p	r	p
Enteroid monolayers	A-to-B	-0.24	0.329	0.07	0.762	-0.30	0.212	0.11	0.668
	B-to-A	-0.11	0.644	0.45	0.056	0.41	0.079	0.09	0.713
	ER	0.18	0.456	0.52	0.021	0.43	0.065	-0.03	0.917
Tissue	A-to-B	-0.11	0.708	-0.45	0.091	-0.48	0.102	-0.08	0.779
	B-to-A	-0.52	0.048	-0.62	0.013	-0.47	0.071	-0.01	0.984
	ER	-0.23	0.462	-0.92	<0.001	-0.02	0.962	-0.13	0.667

Supplementary Table 6: p-values of Wilcoxon signed rank test comparing A-to-B with B-to-A transport direction in enteroid monolayers and tissue for all four substrates Figure 1 and 2

	Enalaprilat	Propranolol	Talinolol	Rosuvastatin
Enteroid monolayers	0.0531	<0.0001	<0.0001	<0.0001
Tissue	0.8945	0.2439	0.001	0.0479

Supplementary Table 7: p-values of Mann Withey t-test comparing apparent permeability in enteroid monolayers with tissue

	Enalaprilat	Propranolol	Talinolol	Rosuvastatin
A-to-B	<0.0001	0.2258	<0.0001	<0.0001
B-to-A	0.0001	<0.0001	0.606	0.5448

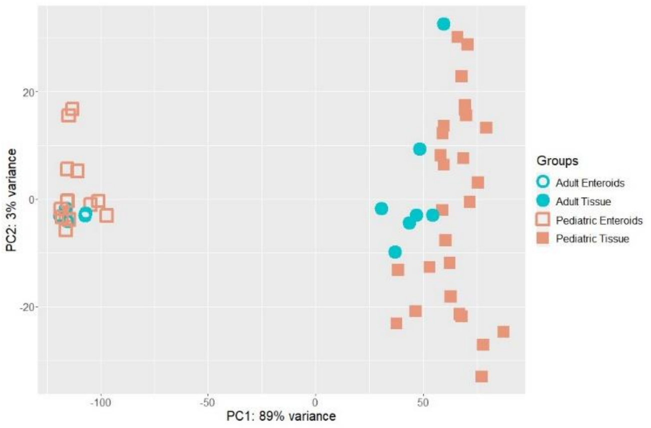

Supplementary Figure 2: Principle component analysis (PCA) of all ileum (pediatric and adult) tissue and enteroid samples included in RNA sequencing. Tissues cluster on the right side n=32 (blue) while enteroid monolayers cluster on the left n=18 (red).

Supplementary Table 8: p-vales for Spearman correlation of gene expression with age measured with RNA sequencing (Figure 6)

	ABCB1		ABCG2		EPCAM		VIL1	
	r	p	r	p	r	p	r	p
Tissue	0.64	<0.0001	0.58	0.0003	0.47	0.005	0.63	<0.0001
Enteroid monolayers	0.23	0.343	-0.028	0.908	0.00	>0.999	0.39	0.099
Correlation tissue vs enteroids	*-0.13*	*0.625*	*-0.12*	*0.646*	*-0.21*	*0.421*	*-0.12*	*0.646*

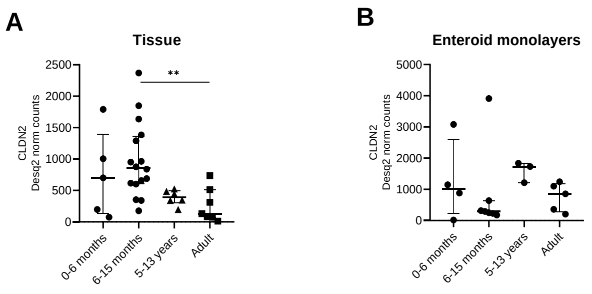

*Supplementary Figure 3: normalized count for barrier marker CLDN2 in tissue and enteroid monolayers. Data represented in median ± IQR. Statistical test: Kruskall-Wallis, **: p=0.001*
